# The Early Presentation of Dementia in People with Down Syndrome: a Systematic Review of Longitudinal Studies

**DOI:** 10.1007/s11065-017-9341-9

**Published:** 2017-03-13

**Authors:** Bianca Alexandra Lautarescu, Anthony John Holland, Shahid H. Zaman

**Affiliations:** Cambridge Intellectual and Developmental Disabilities Research Group, Department of Psychiatry, University of Cambridge, Douglas House, Trumpington Road, Cambridge, CB2 8AH UK

**Keywords:** Down syndrome, Alzheimer’s disease, Dementia, Ageing, Systematic review, Longitudinal

## Abstract

Adults with Down syndrome (DS) are at a very high risk of developing early onset Alzheimer’s disease (AD) due to trisomy of chromosome 21. AD is preceded by a prolonged prodromal “pre-clinical” phase presenting with clinical features that do not fulfil the diagnostic criteria for AD. It is important to clinically characterise this prodromal stage to help early detection of the disease as neuropathology of AD is almost universal by the fifth decade in DS. There is a lack of knowledge of the trajectory of decline associated with the onset of dementia in this population and early signs may be overlooked or misdiagnosed, negatively affecting the quality of life of those affected and the use of early pharmacological or psychosocial interventions. The objective of this systematic review is to evaluate the published literature on longitudinal data in order to identify the cognitive and behavioural changes occurring during the prodromal and early stages of AD in this population. Fifteen peer-reviewed articles met the inclusion criteria, including a total number of 831 participants, with the duration between baseline and follow up varying from 1 year to 47 years. Results suggest that, compared to the general population for which short-term (episodic) memory loss is the most common indicator associated with the onset of AD, in people with DS, executive dysfunction and Behavioural and Psychological Symptoms of Dementia (BPSD) are commonly observed during pre-clinical and early stages and may precede memory loss. The review highlights the importance of using a broad spectrum of assessments in the context of heterogeneity of symptoms. Theoretical and practical implications are discussed, as well as the need for further research.

## Background

Alzheimer’s disease (AD) is a neurodegenerative disorder characterized histopathologically by neuronal death, neuritic plaques and neurofibrillary tangles and clinically by a progressive and irreversible deterioration in cognition and behaviour (McKhann et al. 2011). The greatest prevalence of early onset AD is found in individuals with Down Syndrome (DS), with symptoms appearing before the age of 65 (Hartley et al. 2014) and approximately three-quarters of people with DS over 60 showing clinical evidence of dementia (Lai and Williams 1989). As the life expectancy of people with DS has increased from an estimated mean of 12 years in 1940, to over 60 years now (Bittles and Glasson 2004), the focus of research and of clinical services now includes adulthood and later life.

The later stages of AD in people with DS have been documented and are reportedly similar to symptoms exhibited by people with AD in the general population (Strydom et al. 2010). However, limited information is available with regards to cognitive and behavioural changes prior to diagnosis or during the very early stages (Adams and Oliver 2010). While in the general population the prodromic stage of AD is characterized by impairments in episodic memory, a number of qualitative studies involving people with DS have suggested that the progression of AD in people with DS (DSAD) might be more similar to dementia of the frontal type in the typically developing population, with Behavioural and Psychological Symptoms of Dementia (BPSD) and impairment of executive functions (i.e. goal directed behaviours such as planning, attention, judgement etc.) preceding memory impairments (Deb et al. 2007).

Previous research has also indicated that carers often lack basic knowledge regarding the risk for dementia in this population and are not aware of the symptoms that they should be vigilant for in ageing individuals (Bittles and Glasson 2004). The present paper aims to address this gap in the literature and we report findings from a systematic review of the trajectory of changes that accompany the onset of dementia in this population (i.e. before the formal diagnostic criteria of AD are fulfilled). If there is evidence for an atypical presentation of dementia, such as BPSD and executive dysfunction preceding the development of sufficient symptoms to meet the diagnostic criteria of dementia, this might partially account for the discrepancy between the almost universal presence of neuropathology from age 40 years onwards and the lower (than expected) prevalence rates for clinically diagnosed dementia (Ball et al. 2006) given the prevalence rate of neuropathology. Identifying symptoms that characterize pre-clinical and early AD in people with DS is necessary before further research and clinical interventions, which link findings from neuropathology, putative biomarkers or neuroimaging (Annus et al. 2015) with cognitive features, can be undertaken. Lastly, as new pharmaceutical therapies are developed it is necessary to have reliable measures of performance in all relevant outcome domains so that effectiveness of treatments can be evaluated.

## Methods

**Fig. 1:**
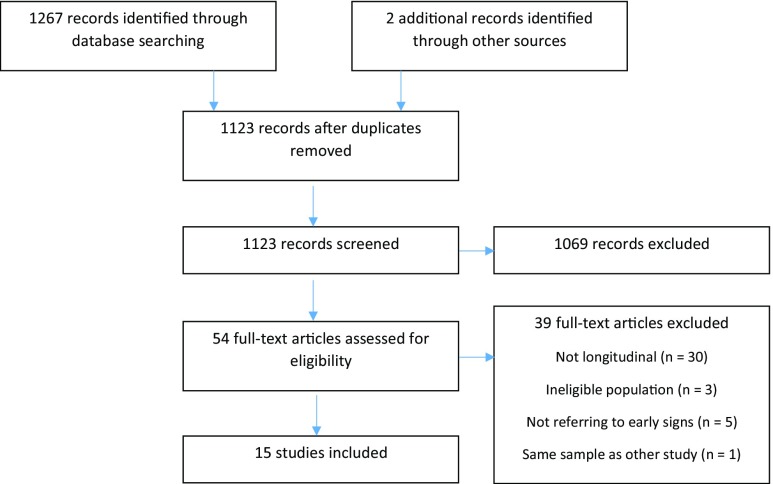
Flow chart showing the process of selecting studies to be included in systematic review

A systematic review was undertaken following the guidelines provided by PRISMA (i.e. Preferred Reporting Items for Systematic Reviews and Meta-Analyses: http://www.prisma-statement.org/) and the Cochrane Collaboration (http://www.cochrane.org/). Potentially relevant studies were identified through searches in citation indexing databases: PubMed (Medline) and PsycInfo, as it has been suggested that they provide broad coverage of biomedical publications worldwide (Suarez-Almazor et al. 2000). Within the electronic database, the search was limited to peer-reviewed journals published between 2000 and 21.01.2015 and included the following terms ((“Down syndrome”[Title/Abstract] OR “Down”s syndrome”[Title/Abstract])) AND (“Alzheimer”[Title/Abstract] OR “Alzheimer”s”[Title/Abstract] OR “dementia”[Title/Abstract]). The search delivered 1267 results and reference lists of selected papers were also searched for potentially relevant studies (Fig. 1).

Titles and abstracts, and then full text articles were reviewed to identify and exclude studies that did not satisfy the previously established criteria: (a) empirical papers, (b) *n* > 1 (c) specified diagnosis of DS, and (d) specific reference to the clinical presentation of early stages of DSAD. Longitudinal studies were chosen as this type of study overcomes the issue of cohort effects and allows a better understanding of the dynamic process of cognitive and behavioural change. To minimize bias, the first author discussed the eligibility and validity of included studies with another member of the research team (AJH), solving any disagreement by consensus.

Moreover, as authors of the included studies have used a large variety of terms to refer to the same concept (i.e. “personality changes”, “behavioural change”, “behavioural excesses and deficits”, “maladaptive behaviour”), we will be using the umbrella term Behavioural and Psychological Symptoms of Dementia (BPSD) in order to enhance the overall consistency of the paper.

## Results

In total, 15 papers reporting on 15 longitudinal studies were selected for data extraction and analysis. Data was collected following guidelines set by the Cochrane Collaboration, with the following variables being extracted: study length, participant number and age at each assessment (mean and standard deviation, if recorded), dementia diagnosis, assessment measures and reported progression of symptoms. In cases where the longitudinal aspect was part of a larger cross-sectional study, data were included only from participants for which at least 2 data points were available. Summary statistics (mean ages and percentages) were calculated where they were not explicitly stated by authors. The duration between baseline and follow up varied from 1 year to 47 years and included a total of 831 participants.

A brief analysis of bias was conducted on the included studies by examining the degree to which the sampling frame is representative of the general population and whether the assessment tools have been validated, as well as the response proportion at both baseline and follow up, with minimal risks of bias being identified by the authors. Table 1 uses Crombie’s Items (Zeng et al. 2015) and the reporting format suggested by the Cochrane Collaboration in order to identify possible areas of bias.

**Table 1:** Risk of bias

	Appropriateness of design to meet the aims	Adequate description of the data	Report the response rates	Adequate representativeness of the sample to total	Clearly stated aims and likelihood of reliable and valid measurements	Assessment of statistical significance	Adequate description of statistical methods	Comment
Ball et al. 2006	+	+	+	+	+	+	+	
Adams & Oliver 2010	+	?	+	+	+	+	+	Insufficient information on diagnosis rates at T1
Kitler et al. 2006	+	+	+	+	+	+	+	
Temple et al. 2001	+	?	+	+	+	+	+	Insufficient information on diagnostic rates at T1
Carr and Collins 2014	?	+	+	−	+	+	+	Insufficient details on diagnostic procedure. All female sample
Cosgrave et al. 2000	+	+	+	+	+	+	+	
Krinsky-McHale et al. 2002	+	+	+	+	+	+	+	
Nelson et al. 2007	+	+	+	+	+	+	+	
Margallo-Lana et al. 2007	−	+	+	+	+	+	+	Different screening procedures used based on availability of information & level of ID
McCarron et al.^2014^	+	?	+	−	+	+		Insufficient information on diagnostic rates at T1. All female sample
Makary et al. 2014	+	+	+	+	+	+	+	
Holland et al. 2000	+	+	+	+	+	+	+	
Devenny et al. 2000	+	+	+	+	+	+	+	
Urv et al. 2008	+	+	+	+	+	+	+	
Määttä et al. 2014	+	+	+	+	+	+	+	

It is also worth mentioning that some of the included studies have overlapping authors (e.g. Kitler et al. 2006; Krinsky-McHale et al. 2002; Devenny et al. 2000; Urv et al. 2008; and Ball et al. 2006; Holland et al. 2000) and since these are longitudinal studies conducted during the same time period, it is likely that some participants overlap within each group, which might impact the overall review.

For each study the following specific aspects were considered: first, evidence for change between time points in scores on cognitive functioning and, secondly, the reported BPSD identified as part of the diagnostic assessment. Where possible, the relationship between these observed changes over time was evaluated. Considering that the length of follow-up, diagnosis reports and assessment methods varied considerably (8 methods of diagnosing dementia, 10 memory tests, 21 instruments for assessing executive dysfunction and 10 measures of behaviour change (Table 2), the results are described through a qualitative synthesis rather than a meta-analysis.

**Table 2:** Variety of assessments included in the systematic review

Abbreviation	Assessment	Developed by/Reported in
AADS	Assessment for adults with developmental disabilities	Kalsy et al. 2002
ABD-Q	Adaptive Behaviour Dementia Questionnaire	Prasher et al. 2004
BEERY VMI	Beery Visual Motor Integration test	Beery and Buktenica 1997
BPVS	British Picture Vocabulary Scale 2nd edition	Dunn et al. 2009
CAMCOG	Cognitive section of CAMDEX-DS	Ball et al. 2004
CAMDEX-DS	The Cambridge Examination for Mental Disorders of Older People with Down’s syndrome and Others with Intellectual Disabilities	Ball et al. 2004
CRT	Cued Recall Test	Buschke 2008
DBC-A	Developmental Behaviour Checklist-Adult	Mohr 2004
DMR	Dementia for Mentally Retarded Individuals	Evenhuis 1990
DLSQ	Daily Living Skills Questionnaire	National Institute of Ageing 1989
DSDS	Dementia Scale for Down Syndrome	Gedye 1995
DSMSE	Down’s syndrome Mental Status Examination	Haxby 1989
EF battery	Tower of London	Krikorian et al. 1994
	Weigl card sorting	Goldstein and Scheerer 1941
	Cats and dogs Stroop task	Gerstadt et al. 1994
	Scrambled boxes	Strauss and Lewin 1982
HBS	Handicaps, behaviour and skills schedule	Wing 1980
IBRMSE	Institute for Basic Research Mental Status Exam	Wisniewski and Hill 1985
LIPS	Leiter International Performance Scale	Leiter 1940
VABS	Vineland Adaptive Behaviour Scale	Sparrow et al. 1984
WRL	Word List Recall	Kittler et al. 2004
SRT	Serial Reaction Time	Buschke 1973
WPPSI-voc	Vocabulary scale of Wechsler Pre-School and Primary Scale of Intelligence	Wechsler 1967
NBAP-D	Neuropsychology Behaviour and Affect Profile	Nelson et al. 2007
Neuropsychological battery	Boston Naming Test	Kaplan 2001
	Wide Range Achievement Test	Jastak and Wilkinson 1984
	Verbal fluency	-
	BPVS	Dunn et al. 2009
	Fuld Object-Memory test	Fuld 1981
	Visual Motor Integration Test	Beery and Buktenica 1997
	WISC-III	Wechsler 1974
	WPPSI	Wechsler 1974
	20-hole foam pegboard VABS	- Sparrow et al. 1984
PPVT-4	Peabody Picture Vocabulary Test 4th Edition	Dunn 2007
RBMT-C	Rivermead Behavioural Memory Test for Children	Wilson and Ivani-Chalian 1995
RSMB	Reiss Screen for Maladaptive Behaviour	Reiss 1994
S-R paradigm	Simple visual discrimination, reversal learning, Delayed non-match to sample, landmark	Nelson et al. 2007
TSI	Test for Severe Impairment	Albert and Cohen 1992
WISC-R	Wechsler Intelligence Scale for Children-Revised	Wechsler 1974

### Presentation and Progression of Dementia in the People with DS

In the general population, mild cognitive impairment (MCI) is regarded as the prodromal stage of dementia, but it has been argued that, in the case of people with DS, the exact hierarchy of loss of function is very difficult to establish (Cosgrave et al. 2000). Out of the 15 studies identified here, which attempted to address this question, nine suggested that ‘frontal-like symptoms’ were the earliest sign of AD in this population. Only two studies reported memory as the earliest sign of dementia. Out of the remaining four studies, two reported no decline that could not be accounted for by normal ageing and two did not report the order in which domains of cognitive functioning were affected.

The studies reporting the progression of clinical signs of DSAD are summarized in Table 3 and further discussed in the following sections. It is important to note that the majority of studies included in this review (with the exception of Määttä et al. 2014) have used various screening tools, rather than a diagnosis made by a clinician. Thus, it is possible that discrepancies between results are due to inadequate classification of participants with or without dementia, as well as due to the use of different diagnostic methods in each individual study (Ballard et al. 2016).

**Table 3:** Studies included in the systematic review

Study and sample characteristics^a^	Diagnosis/Screening tool	Diagnosis T1% changes and mean age (SD)^b^		Diagnosis T2% changes and mean age (SD)		Assessments^c^	Progression
Ball et al. 2006 *n* = 52 T1 to T2 = 5 years Mean age = 48.3 (range 36–72)	CAMDEX-DS	Healthy (38.1%) BPSD (34.5%) DFT^d^ (20%) AD (7.2%)	43.0 (9.0) 41.3 (7.2) 44.9 (8.6) 49.5 (2.5)	Healthy (23%) BPSD (34.6%) DFT (23%) AD (19.2%)	49.8 - 46.0 (6.8) 44.8 (5.8) 55.8 (5.5)	Memory (CAMCOG) *EF and L* (CAMCOG) *BPSD and ADL* (CAMDEX-DS)	Progression from early BPSD to characteristics associated with frontal lobe dysfunction, prior to the development of AD. 44% of those with BPSD progressed to DFT and AD (compared to 5.6% without). 36% of those with DFT progressed to AD (compared to 5.4% without).
Adams and Oliver 2010 *n* = 30 T1 to T2 = 1.3 years Mean age = 44.5 (range 34–64)	-	- - - -	- - - -	No decline (66%) Deterioration (33%)	42.3 (6.8) 48.9 (7.2)	Memory - *EF and L* (NAID, EF battery, BPVS) *BPSD and ADL* (AADS, VABS)	Participants whose cognitive functions deteriorated between T1 and T2 showed behavioural excesses and impaired EF. There was no formal diagnosis procedure.
Kitler et al. 2006 *n* = 42 T1 to T2 = 3 years Mean age = 44.3 -	Caregiver reports	0%	-	0%	-	Memory (WLR,CRT, SRT) *EF and L* (WLR) BPSD and ADL -	Verbal intrusions at T1 predicted memory changes at T2. None of the participants met criteria for AD.
Temple et al. 2001 *n* = 35 T1 to T2 = 3 years Mean age = 46 (range 29–67)	DSDS	- - - - - - - -	- - - - - - - -	Healthy (51.4%) Possible AD (22.8%) Early AD (20%) AD (5%)	42 - 48.8 - 53.1 - 55 -	Memory (BNT, FOME, VMI) EF and L (Battery) BPSD and ADL (VABS, Caregiver reports)	A higher level of cognitive functioning predicted less decline on measures of EF.
Carr and Collins 2014 *n* = 29 T1 to T2 = 47 years Mean age = 47 -	-	Healthy (100%) Dementing (0%)	- - - -	Healthy (75%) Dementing (24%)	47 - 47 -	Memory (RBMT-C) EF and L (NAID,HBS, BPVS, WPPSI-voc) BPSD and ADL -	All scores showed marginal decline, possibly reflecting normal ageing. There was no formal diagnosis procedure. Only 2/7 dementing participants had diagnosis confirmed by clinician.
Cosgrave et al. 2000 n = 71^e^ T1 to T2 = 5 years Mean age = 51.8 (range 40–76)	Checklist of symptoms	Healthy (91.3%) AD (8.7%)	- - - -	Healthy (56.3%) AD (43.7%)	- - - -	*Memory* (TSI) EF and L (TSI) *BPSD* *-* *and ADL* (TSI, DLSQ)	Memory problems were the earliest sign of AD regardless of ID level
Krinsky-McHale et al. 2002 *n* = 85 T1 to T2 = 3 years Mean age = 48.5 -	DSDS	No AD (100%) AD (0%)	44.27 (6.8) - -	No AD (83%) AD (16.4%)	47.4 (6.6) 54.48 (6.3)	*Memory* (SRT) EF and L - BPSD and ADL -	Memory (long term storage and retrieval) was affected in early-stages of dementia, preceding reported behavioural symptoms by more than a year.
Nelson et al. 2007 *n* = 19 T1 to T2 = 1 year Mean age = 40 (range 24–55)	DMR	Healthy (61%) AD (39%)	- - - -	Healthy (48%) AD (52%)	39.33 (8.6) 41.58 (8.7)	Memory (DNMTS) *EF and L* (WAIS-III, S-R Paradigm) *BPSD and ADL* (NBAP-D)	Fronto-temporal measures predicted dementia status. These results suggest decline in functions other than memory early on in the course of AD. Scores did not decrease significantly over the course of 1 year.
Margallo-Lana et al. 2007 *n* = 64 T1 to T2 = 15 years Mean age = 39.1 -	Various procedures ^f^	Healthy (94%) AD (5.4%)	- - 58.4 (1.5)	- - AD (21%)	- - 59.^g^ (9.6)	Memory - EF and L (PCFT) BPSD and ADL (VABS)	Deterioration in cognitive function was observed only for participants with mild ID. No changes in personality or behaviour reported.
McCarron et al. 2014 *n* = 77 T1 to T2 = 14 years - >35	ICD-10, DMR	- - - -	- - - -	Healthy (10.4%) AD (89.6%)	- - 55.4^h^ (7.1)	Memory - *EF and L* (TSI, DSMSE) BPSD and ADL (DLSQ)	Decline on TSI and DSME occurred 1 year prior to diagnosis. Decline on DLSQ occurred over 3–4 years prior to diagnosis and on DMR over 5 years
Makary et al. 2014 *n* = 28 T1 to T2 = 4 years Mean age = 37.7 (range 28–56)	-	0%	-	0%	-	Memory - EF and L (PPTV-4) BPSD and ADL (ABD-Q, DBC-A)	No decline was reported on measures of personality and behaviour
Holland et al. 2000 *n* = 68 T1 to T2 = 1.5 years Mean age = 42.3 -	CAMDEX-DS	Healthy (75.7%) AD (24.3%)	- - - -	Healthy (61.8%) AD (38.2%)	48.6 (6.0) 52.9 (7.5) 56.7 (6.7) 53.1 (5.7)	Memory (CAMDEX-DS) EF and L (CAMDEX-DS) *BPSD and ADL* (CAMDEX-DS)	Initial changes reported in BPSD and reflected subsequent diagnosis. BPSD were reported more (46.67%) than memory issues (9.33%). BPSD without memory issues were more commonly reported in younger participants.
Devenny et al. 2000 *n* = 68 T1 to T2 = 10 years - -	DSDS	Healthy (100%) AD (0%)	- - - -	Healthy (64%) Questionable (14.7%) Early (7.3%) AD (10.2%)	48.6 (6.0) 52.9 (7.5) 56.7 (6.7) 53.1 (5.7)	Memory (CRT,SRT) *EF and L* (WISC-R) BPSD and ADL -	Sequence of cognitive decline involved in progressively more areas of EF and the number of areas affected was associated with the severity of dementia.
Urv et al. 2008 *n* = 138 T1 to T2 = 1.5 years Mean age = 53.4 -	DMR	Healthy (64%) Questionable (20.7%) Possible (5%) AD (9.5%)	51.6 (5.7) 57.7 (7.8) 61.1 (7.3) 59.1 (8.1)	Non-converters (73%)^i^ Converters (27%)	48.6 (6.0) 52.9 (7.5) 56.7 (6.7) 53.1 (5.7)	Memory (SRT) EF and L (DMSE, IBRMSE, Vb fluency, BEERY VMI, WISC-R, TSI) *BPSD and ADL* (VABS, RSMB)	Individuals transitioning into the early stages of dementia displayed increased maladaptive behaviours.
Määttä et al. 2014 *n* = 25 T1 to T2 = 3 years Mean age = 48.8 (range 25–65)	Clinical evaluation	No AD (40%) AD (60%)	40 - 49.7 -	No AD (40%) AD (60%)	43.1 - 52.9 -	Memory - EF and L - *BPSD and ADL* (VABS)	Behaviour scores declined significantly during the course of the study and were associated with AD.

^a^n refers to the number of participants as recorded at T2 (years)

^b^All percentages are expressed in terms of number of surviving subjects

^c^Italics are used to emphasize the domain in which changes were reported first

^d^Authors refer to this stage characterized by executive dysfunction as “Dementia of frontal type (DFT)

^e^All-female population

^f^In the study conducted by Margallo-Lana, dementia diagnosis was made in different ways depending on the information available and the level of ID (e.g. caregiver reports, ICD-10, medical records)^g^ Age of death is reported. 92 participants (5 AD) are part of the original cohort, 87 enter study (14 pass away with AD, 9 without AD) and 64 survive (4 AD)

^h^Average age of diagnosis is reported, rather than age and percentages at T1 and T2. 58% of the sample had passed away by T2

^i^Participants who progressed or not from no dementia to a “questionable” status

### Memory

DS is known be associated with impairments in working memory, specifically remembering information for short periods of time (Silverman 2007). It has been suggested that tasks that rely on the hippocampus (e.g. associations of items in space and time) are commonly impaired in this population, hence making difficult the measurement of decline in these domains as a marker of AD-related change. By taking into account the level of baseline functioning, researchers interested in memory decline in people with DS have mostly focused on verbal list learning and scene learning (Sabbagh and Edgin 2016).

Compared to the general population where episodic memory impairments characterize prodromal stages of AD in the majority of cases, only two of the longitudinal studies included in this review identified impairments in memory as one of the earliest symptoms of DSAD (Krinsky-McHale et al. 2002; Cosgrave et al. 2000). Krinsky-McHale et al. (2002) found that decline in memory function scores preceded the onset of global changes associated by the authors with the onset of dementia, as reported in informant interviews. Decline in memory function preceded more global changes by more than a full year and in some cases by up to 3 years. More specifically, verbal explicit memory was one of the first domains that were affected, with individuals’ scores on the Selective Reminding Test declining by 20% on two consecutive test sessions, The authors compared the results of participants with dementia to those of age- and IQ-matched non-demented individuals with DS and concluded that the dramatic declines observed in DSAD were distinguishable from normal ageing pattern. Interestingly, even in cases where participants were capable of performing the task, those in the early stages of dementia showed deficits in their capacity to encode and retrieve information from long-term memory.

This view was supported by a previous study in which memory loss was one of the first identifiable hallmarks of AD in an all-female sample of older adults with DS (Cosgrave et al. 2000). In this study, participants had difficulty remembering items that they had seen as part of a previous task (i.e. delayed memory) and difficulty remembering in which hand a paper clip was held by the examiner after both hands were placed behind the examiner’s back (i.e. immediate memory).

However, these findings were only applicable in the case of individuals with mild (IQ 55–70) and moderate ID (IQ 40–55), and memory problems were more difficult to identify in participants with more severe intellectual disabilities (severe = IQ 25–40, or profound = IQ < 25) due to floor effects on established tests. More specifically, participants with IQ under 40 had very low scores on memory tests regardless of dementia diagnosis and thus, these measures may not be suitable as an indicator of decline over time in this population. More recently, in the DSM-V, IQ test scores have been removed from the diagnostic criteria of ID (American Psychiatric Association 2013), emphasizing the need for a more comprehensive assessment in which severity of ID will be based on the level of adaptive functioning, rather than IQ, as the former determines the level of support required (Oakley et al. 2003).

In the study conducted by Devenny et al. (2000), authors suggested that decline does not occur globally but rather as a systematic and progressive loss of cognitive functions, with memory loss being a predominant symptom in all participants who also showed decline on the Block Design and Coding subtests of the WISC-R. These participants were classified as having “questionable decline”. However, the authors emphasize the fact that none of these participants had received an official diagnosis from a physician and thus advise caution when interpreting their results, as it is possible that the observed declines were related to some other undetected condition.

A different view, however, is that decline in memory scores (even in the presence of developmental memory impairment) are observed in all ageing individuals with DS, regardless of dementia status (Ball et al. 2006; Devenny et al. 2000; Carr and Collins 2014). It is possible that by examining alternative measures of decline in other cognitive domains, the generalized pattern of deterioration that accompanies old age can be distinguished from preclinical AD. In the study conducted by Ball et al. (2006), informant reported memory changes were not correlated with subsequent diagnosis of AD and all participants who showed changes in memory also showed BPSD. However, in people with DS, early changes in memory may be more difficult to notice compared to BPSD, the latter having a greater impact on the individual’s daily life (Adams and Oliver 2010). Certain learning and memory tasks such as cued-learning and recall tasks may prove to be of a greater sensitivity and specificity for distinguishing between AD and non-AD in this population (Benejam et al. 2015).

More recently, a review by Sabbagh and Edgin (2016), suggested that the earliest symptoms of dementia in individuals with DS are often subtle but are firstly evident in BPSD rather than episodic memory. They are thus arguing that the late diagnosis of DSAD is often due to the fact that current classification systems have their emphasis on memory impairments, having been modelled after the diagnostic criteria of dementia in the general population and are unfit for use in the DS population (Nieuwenhuis-Mark 2009).

### Executive Function and Language

Functional impairments in executive functioning (executive dysfunction), as referring to informant reported difficulties with various goal-directed behaviours, such as planning and attention, and also performance on specific cognitive tests, have been identified in the majority of selected studies as preceding memory problems in adults with DS. However, in the studies reviewed there was not obvious overlap in terms of specific areas of decline identifed by repective authors. In Ball et al. (2006), the most commonly reported changes were impairments in planning, attention and lack of foresight, while in another study (Adams and Oliver 2010), the most affected areas were working memory, agnosia, aphasia and apraxia, with cognitive deterioration not being solely attributable to memory issues. In the Ball et al. (2006) paper, a version of the CAMCOG neuropsychological test battery modified for use with people with DS was completed at baseline and follow up assessments, to provide a measure of decline in global cognitive function. To provide more specific information on the sequence of decline in “frontal lobe associated EF” over the 5 years prior to diagnosis, an additional measure labelled “EF and attention” was designed by combining CAMCOG scores for abstract thinking, attention-calculation, verbal fluency and the clock drawing item. However, impairments in planning and foresight were measured through informant reported changes, rather than a more objective measure. Given the variety of living situations of participants in this study [i.e. residential homes (82%), sheltered accommodation (4%), nursing homes (2%), with a parent orrelative (13%)], we advise caution when interpreting these results. The caution is based on research showing that informant reports differ both qualitatively and quantitatively when recorded from personnel in an institution compared to parents (Nieuwenhuis-Mark 2009). Moreover, it is possible that “lack of foresight” could be affected by intellectual ability and this should also be taken into consideration, as 41 out of 55 participants in this study had moderate or severe learning disability.

In one of the studies, executive dysfunction was investigated by looking at verbal intrusions as an indicator of disruption of inhibition control (Kitler et al. 2006). Responding with an irrelevant word during a task of verbal memory retrieval was found to be predictive of performance on two out of three memory tasks administered within the next three years. Therefore, the authors argued that verbal intrusions are an early sign of Alzheimer-related neuropathology, preceding declines in memory. Interestingly, middle aged participants with DS made more verbal intrusions at baseline compared to participants with unspecified ID (74% vs 44%), giving further support to the idea that executive functioning is more sensitive to decline in DS than in other populations.

Another component of EF was examined by Nelson et al. (2007), who identified the NBAP Pragnosia scale as useful in classifying 80% of all dementia cases. The authors argued that impaired pragmatic language function represents an aspect of cognitive decline, as this measure appears to be strongly correlated with the assessment of dementia status (DMR Cognitive Scale). These impairments appeared to be most common in younger subjects, suggesting decline in functions other than memory, early in the course of dementia. More recently however, researchers have argued that verbal scores do not show significant decrease over the course of 47 years (Carr and Collins 2014), but these results might represent a sample bias, as the stronger representation of verbally able women might have influenced the overall stability of verbal scores in the group.

However, the exact sequence in which subdomains of executive functioning are affected has not yet been established. This could be largely due to the heterogeneity of DS phenotypes. Devenny et al. (2000) attempted to answer this question by following up participants over a 10 year period and noting that scores which showed decline early in the disease process were those that required perception of abstract stimuli and visuo-motor coordination. As participants progressed from prodromal stages to early dementia, deficits in comprehension, measures of visuospatial organization and the working memory component of language were observed, followed by vocabulary, information and digit span tasks in the middle stages of AD. As decline associated with the development of AD seems to involve progressively more areas of cognition and the number of areas in which decline was observed of was correlated to the severity of dementia, the authors conclude that cognitive decline in AD in people with DS is not global, but follows a predictable sequence. Executive functioning seems, according to the evidence presented above, to be affected in pre-clinical or early stages of AD and thus, detecting these changes could prove to be helpful in predicting a later diagnosis of AD.

### Behavioural and Psychological Symptoms of Dementia (BPSD)

Behavioural and Psychological Symptoms of Dementia (BPSD) have been defined as a “heterogeneous range of psychological reactions, psychiatrics symptoms and behaviours resulting from the presence of dementia” (Finkel 2001). Recent literature has suggested that BPSD are reported in the prodromal and early stages of DSAD and might predict a more severe diagnosis. Often, BPSD are reported in the absence of informant observed functional memory decline (Ball et al. 2006), in contrast to the presentation of AD in the general population, where impairments in episodic memory are commonly the first reported changes.

The hypothesis that BPSD and executive dysfunction precede memory issues in the pre-clinical and early stages of AD in DS is relatively recent (Ball et al. 2006; Adams and Oliver 2010; Kitler et al. 2006; Nelson et al. 2007; Urv et al. 2008; Määttä et al. 2014; Holland et al. 2000). This systematic review indicates that there is a limited but significant amount of research supporting the hypothesis, indicating that people with DS exhibiting BPSD (i.e. those with a history of the onset of these behaviours as opposed to such behaviours being lifelong) are more likely to decline functionally and on testing have evidence of impaired executive functioning compared to those with no such changes (44% compared to 5.6% of those without BPSD). Later the impaired group are then more likely to exhibit memory problems and meet diagnostic criteria for dementia (Ball et al. 2006). Similarly, in the study conducted by Holland et al. (2000), younger people with DS, who did not yet meet the full criteria for a clinical diagnosis of AD, exhibited more ‘frontal-like symptoms’, while older people had higher rates of clinically diagnosed AD. These results were supported by Nelson et al. (2007), whose study, although not identifying any significant decline in function, due to the short duration between baseline and follow up, argued for the high reliability and validity of measures of “frontal-like symptoms” in the detection of AD early in the disease process, suggesting that EF and BPSD may indeed precede or accompany memory changes in this population

This view is also supported by the results of Holland et al. (2000). At the time of an initial assessment more participants were reportedly exhibiting changes in behaviour (46%) than changes in memory (9%), whereas at follow up. Older participants had the largest proportion of reported deteriorations in memory and personality, whereas younger participants showed high rates of BPSD in absence of any memory issues, suggesting a trend of decline from behaviour and personality to a combination of domains.

This sequential progression of BPSD between various stages of dementia was further examined in studies that reported correlations between the severity of BPSD and a dementia diagnosis (Määttä et al. 2014; Urv et al. 2008). If indeed BPSD are an indicator of dementia development, the question that arises is whether certain types of behaviours are more likely to develop. In Urv et al. (2008), participants who showed cognitive decline insufficient for a diagnosis of AD exhibited more BPSD directed towards others (i.e. destructiveness, aggression) than those with a diagnosis of AD, who exhibited more BPSD directed towards the self (i.e. fearfulness, lack of energy, withdrawal, sadness and self-injury). Interestingly, participants who progressed from no diagnosis to questionable dementia were more likely to experience a worsening of scores on certain measures (sadness, increased dependency, confusion, fearful and regressive behaviour and social inadequacy), while the scores of people who were dementia-free at T2 remained relatively stable on these measures (Urv et al. 2008). Contrary to these findings, Adams and Oliver (2010) and Makary et al. (2014) suggested a more positive pattern of ageing than previously reported.

Similarly to the situation described in the case of executive functioning, a specific pattern of deterioration in BPSD subdomains is difficult to identify. It was proposed that some of the earliest signs of dementia in people with DS are observed in language and socialization difficulties (Margallo-Lana et al. 2007).

Improving our understanding of BPSD and their relationship to AD is essential, given that there are people with DS with very low levels of cognitive functioning who are unable to be diagnosed by measures of cognition alone due to floor levels. This group could, however, be diagnosed on the basis of BPSD and everyday skills (Margallo-Lana et al. (2007). Similar conclusions were reached by Cosgrave et al. (2000), who concluded that even though in their study memory seems to be the earliest symptom, severe intellectual disability conceals the symptoms and thus, BPSD might be easier to detect

Informant reported BPSD such as social withdrawal, apathy (Ball et al. 2006; Holland et al. 2000), inactivity and lack of interest (Adams and Oliver 2010), have been reported in all stages of DSAD (Urv et al. 2008) and it is likely that they significantly affect activities of daily living, with declines having been reported in domestic activity, self-direction and responsibility (Määttä et al. 2014). However, the authors note that depression is one of the factors that correlate most strongly with such changes (Urv et al. 2008) and may often act as a confound due to diagnostic overshadowing (Määttä et al. 2014), alongside hypothyroidism. Because of this potential confound, caution is advised when discussing the role of BPSD in the progression of DSAD and these reported changes must always be analyzed as part of a wider context.

Changes such as restlessness, aggression, repetitive speech and being uncooperative were most often reported in pre-clinical and early-stages of AD and thus might represent some of the earliest observable signs of dementia (Adams and Oliver 2010). Observation of these signs seems to be more likely to lead to a referral for dementia assessment and were shown to emerge alongside cognitive decline. In contrast, Carr and Collins (2014) in her long-term follow-up of a birth cohort did not find any increase in aggression with the development of dementia.

Other BPSD, such as emotional lability, lack of concern for other people, stubbornness, disinhibition and impulsivity, were also reported prior to a diagnosis of dementia (Ball et al. 2006). Stubbornness was the second most reported BPSD after apathy in a sample of aging people with DS (Holland et al. 2000), and increases in BPSD were identified as successful predictors of dementia status. When analyzed alongside scores for pragnosia, BPSD successfully predicted over 70% of dementia cases (Nelson et al. 2007). Urv et al. (2008) suggests that these changes are present before a dementia diagnosis and gradually worsen as dementia progresses, until late stages of AD when the number of reported distinct behaviours is reduced.

#### Activities of Daily Living

Activities of daily living (ADLs) represent a number of activities such as continence, eating, walking or grooming (Ward et al. 1998) and have been shown to be a good predictor of hospital admission and mortality in the elderly population (Ferrucci et al. 1997). Research has suggested that an indicator of the development of AD in people with DS is the loss of everyday skills and difficulties with activities of daily living (Margallo-Lana et al. 2007), significantly more affected at age 47, compared to age 30 (Carr and Collins 2014). This pattern of decline seems to be supported by other research (Cosgrave et al. 2000) and it has thus been suggested that investigating ADLs could be very valuable in diagnosing DSAD, especially in people with severe ID, for whom other changes might be difficult to notice (Margallo-Lana et al. 2007). However, caution is advised when interpreting these results, as gender has been suggested to influence rate of decline in ADL in the general population (Ward et al. 1998) with women maintaining skills for a longer time in areas such as food preparation, housekeeping and laundry (Lawton and Brody 1969).

Regarding the hierarchy of loss of ADLs, Cosgrave et al. (2000) noted that loss of independence in personal hygiene was among the first signs of dementia, alongside spatial disorientation. The last skills to be lost were eating and shaking hands, with 86.9% of participants being able to perform this latter task even in later stages of AD. This is not surprising given the high baseline for the performance of this behavior (i.e. at the beginning of the study, 100% of participants with moderate ID were able to shake hands with the examiner). However, at the end of the study, many participants were completely dependent on nursing staff, having many other difficulties such as incontinence and severe motor difficulties. Considering the advanced nature of the disease in these participants, it is difficult to pinpoint whether loss of a certain skill is due to loss of social conventions or impaired motor function. Regardless, more recent studies in the general population do suggest that motor skills should be addressed in interventions designed for people with AD in order to improve their daily living (Oakley et al. 2003).

## Discussion

### Early Signs of Dementia in DS

This review highlights the importance of using a broad spectrum of assessments when examining the sequence of decline of dementia in people with DS, particularly in the early stages during which the presentation of dementia may differ between individuals. While there is no overall consensus, there are a majority of research studies identifying clinical characteristics, other than the development of functional memory changes that occur early in the course of AD. However, an evaluation of the papers included in this systematic review indicates that assessing the hierarchy of decline that accompanies the development of dementia in DS is a challenge. First, from a methodological perspective, change has been identified in two separate ways, one being a structured informant-based interview asking specifically about functional changes and the other being the use of established cognitive tests that can be repeated over time to established whether there has been a change in test scores or not. Secondly, as the diagnosis of AD represents a threshold effect, it is difficult to pinpoint in time the exact onset of symptoms. Thirdly, heterogeneity of symptoms seems to be characteristic of the early stages, and inferences regarding which subdomains of functioning are preferentially affected are very difficult to make. Fourthly, in people with DS it is especially difficult to determine whether the observed symptoms are due to age-related changes in this population or to preexisting cognitive impairments (Ball et al. 2006). In the longest longitudinal study included in this review, scores on the majority of cognitive tests declined by age 47, regardless of dementia status (Carr and Collins 2014), emphasizing that researchers must be cautious when interpreting results, as for a proportion of participants showing this decline it might suggest pre-clinical symptoms of dementia, it might also reflect normal ageing.

The findings from this systematic review would indicate that BPSD and impairments in executive functioning mark the early course of DSAD. Numerous components of executive function and behavior seem to be affected before memory skills begin to decline, suggesting that current diagnosis procedures of AD in DS may not be effective until later stages. This view is supported by a recent review (Ballard et al. 2016), which suggested that dementia in DS initially manifests as BPSD, only to later be followed by changes in cognition.

There are, however, important differences between study findings that may reflect genuine sample differences or may be accounted for by differences in methodology. These differences make an overall picture difficult to ascertain. BPSD and executive dysfunction were often investigated as part of studies primarily concerned with cognition whereas memory changes were reported as part of studies that were carried out with the intention of confirming the presence of memory decline. Moreover, some studies only analyzed a restricted range of abilities and reported on total scores alone, without specifying the exact area of decline. Given the wide variety of screening and diagnosis tools in use, as well as recent research suggesting decline in cognitive function regardless of whether participants have had a formal diagnosis of dementia, we support the recent suggestion made by Ballard et al. (2016), that measures of prevalence reported in published papers should be interpreted with a degree of caution.

Moreover, as with all neuropsychological testing, there is overlap in measurement. For example, a decline in a test of episodic memory would have to be carefully interpreted if there are poor results on attentional tasks. Due to this confounding factor, we advise caution when discussing the results of these papers, as it often is difficult to establish whether a participant’s decline on one measure is not influenced by decline in another related domain.

Lastly, interpreting results was hindered by the fact that the majority of studies did not report on the level of intellectual disability of participants. This issue is essential, considering that the salience, manifestation and degree of impairment determined by the emergence of AD symptoms can vary according to the level of premorbid intellectual capacity and also the person’s ability to compensate for the newly acquired deficits. Margallo-Lana et al. (2007) demonstrated that significant cognitive decline at follow - up could only be identified for cases where the baseline score had been at or above the median (mild and moderate ID), with little changes being noticeable for people with severe and profound ID. Moreover, the majority of studies (Adams and Oliver 2010, Kitler et al. 2006, Carr and Collins 2014, Cosgrave et al. 2000, Krinsky-McHale et al. 2002, Nelson et al. 2007, Margallo-Lana et al. 2007, McCarron et al. 2014, Makary et al. 2014, Määttä et al. 2014) did not document whether dementia was at an early or advanced stage. As changes in function seem to vary both quantitatively and qualitatively over the course of AD, future research should report the stage of decline, as well as age and IQ.

### Recommendations

Given the findings of this review, we believe that establishing a baseline level of cognitive functioning and behaviour in people with DS would be extremely useful in following up on changes in domains known to be affected by AD. We therefore advocate the development of a set of common measures appropriate for individuals with DS that would include in depth evaluation of memory, executive function, language and behaviour and that can be used both in clinical settings as well as research. This would allow the implementation of Burt and Aylward’s model of best practice (Burt and Aylward 2000) which suggests establishing a baseline of premorbid functioning before the age of 35, having annual reassessments and conducting a detailed diagnosis in cases where decline is evident. More research needs to be conducted in order to decide on the exact measures to be included in this tool set, given the intra-individual variability in task performance in people with DS (Krinsky-McHale et al. 2008). At present, standard tools developed for the assessment of cognition in the general population often show floor effects when administered to individuals with DS (Stanton and Coetzee 2004) and the majority of the assessment tools are based on the premise that respondents have an IQ in or close to the normative range and require other skills such as good verbal skills, adequate attention or dexterity (Prasher et al. 2004). Burt et al. (2005) have emphasised the need for an appropriate scoring method for diagnosing dementia in this population and suggested that slope scores are more useful than difference scores. For example, institutionalised individuals with DS are known to score lower on neuropsychological tests than people living in the community (Nieuwenhuis-Mark 2009) and thus having an established baseline and measuring change over time in a longitudinal follow up design would be a better approach than cross-sectional studies.

Given that the majority of the studies included in this review point towards BPDS as an early sign of dementia in DS, we believe that there is an urgent need for the development of an acceptable, validated and standardised evaluation scale for BPSD in DS (Sinai et al. 2016), similar to BEHAVE-AD in the general population (Dekker et al. 2015).

Furthermore, diagnostic and assessment measures developed for use in the general population are often used in studies investigating AD in DS, which often introduces confounding factors (Ballard et al. 2016). This issue was pointed out by Dekker et al. (2015) who stated that although more than 20 scales have been developed to assess BPSD in AD in the general population (Finkel 2001), none of these have been validated in people with DS and therefore are not equally valuable for use in this population as they do not take into consideration factors such as pre-existing behavioural issues associated with ID. In line with the findings of Dekker et al. (2015), such a scale could be implemented in routine practice alongside existing assessment procedures which monitor change over time, as well as being used in longitudinal research and clinical trials.

Having comprehensive assessment tools for outcomes in clinical trials would enable a targeted approach to drug development, which would be essential given recent findings suggesting that people with DS respond differently to treatment when compared to late-onset AD typically developing controls (Ballard et al. 2016). Moreover, a better understanding of BPSD in DS would increase acceptance from the perspective of a carer who would understand that behaviour they perceive as “challenging” is not deliberate but may be a symptom of dementia (Dekker et al. 2015). Imporved recognition of symptoms would facilitate access to non-pharmacological treatments such as behavioural and music therapy and psychosocial interventions (Gauthier et al. 2010).

To account for the fact that BPSD and executive dysfunction seem to be affected earlier than episodic memory in the course of dementia, we suggest Mortimer’s reserve capacity hypothesis (1981) as a viable theoretical explanation. According to this model, the frontal lobes of people with DS are underdeveloped and thus a smaller buden of neuropathology in the brain may be required to reach the threshold at which functioning is impaired. Volume reduction of frontal lobes has been observed during childhood, while hippocampal volume reduction has only been reported later in life (Jacola 2012). These observations would account for the preferential decline in areas of cognition sub-served by these areas. Indirect support for Mortimer’s reserve capacity hypothesis comes from the longitudinal study conducted by Temple et al. (2001), in which progression of dementia appears to be slowed by having high levels of pre-morbid cognitive functioning. The authors suggest that environmental interventions such as improving the level of education and number of years in employment might represent successful strategies to reduce cognitive decline. The suggestion that early clinical presentation of AD in DS matches that of frontal-based symptoms can serve as a theoretical foundation for the findings of neuroimaging studies such as the recent amyloid PET imaging with 18F–FDDNP study that showed increased frontal predominance of amyloid pathology in people with DS who had a diagnosis of AD, compared to typically developing patients with late-onset AD (Nelson et al. 2011).

To conclude, even though there is no overall consensus on what represents the earliest observable sign of dementia in DS, this review highlights the heterogeneity of affected domains. BPSD, developing for the first time in later adult life, predicts the subsequent development of dementia in the majority of the included studies. There is also evidence that emergence of BPSD is underpinned by impairments in executive functioning that may implicate impairments in frontal lobe integrity and in related brain networks (Ball et al. 2008). We recommend that future research should investigate a wider variety of processes, to reflect the heterogeneity of domains that seem to be affected in the prodromal and early stages of dementia in this population. Moreover, considering that in the general population amnestic MCI is widely accepted as the prodromal stage of AD, an alternative should be proposed for people with DS, to reflect this difference in progression. The key to early diagnosis is knowing what observed changes should raise the index of suspicion sufficiently to indicate the need for a full diagnostic assessment and cognitive evaluation. The findings of this review are especially relevant given that current diagnosis procedures (ICD-10 and DSM-V criteria) emphasize declines in memory functions and therefore in this population may fail to facilitate the early detection and diagnosis of dementia when it does occur.

Acknowledgments

This systematic review is part of a larger research study funded by the Baily Thomas Charitable Fund, Addenbrookes Charitable Trust and the Health Foundation. Queens’ College Cambridge contributed via a grant which enabled the dissemination of these findings at BNA 2015 Festival of Neuroscience in Edinburgh.
